# Intrawound Vancomycin Powder in Posterior Lumbar Fusion: In Vitro Osteoblast Effects and Clinical Outcomes in a Historical-Control Cohort

**DOI:** 10.1177/21925682261507654

**Published:** 2026-11-11

**Authors:** Heili Truumees, Lindley I. N. Friedman, Joshua Schmirler, Sarina Sinclair, Zachary Funk, Danielle M. Geerling, Nicholas T. Spina, William Ryan Spiker, Darrel S. Brodke, Brandon D. Lawrence, Brian A. Karamian

**Affiliations:** 1 Department of Orthopaedics, 7060 University of Utah, Salt Lake City, UT, USA

**Keywords:** Intrawound Vancomycin, Surgical Site Infections, Pseudoarthrosis, Posterior Lumbar Fusion, Complications

## Abstract

**Study Design:**

Retrospective analysis.

**Objectives:**

To analyze the immediate post-operative effects on osteoblast activity, and long-term impact on bone healing, of intrawound vancomycin powder following posterior lumbar fusion (PLF).

**Methods:**

This study had two phases: (1) an in vitro analysis of vancomycin effects on osteoblasts and (2) a retrospective chart review—spanning over a decade—analyzing outcomes of vancomycin use in PLF patients. Cellular metabolic and osteogenic activities were evaluated using well-established in vitro assays. The retrospective chart review included patients ages 18+ who underwent PLF (four levels or fewer) between January 2007-December 2015. Patients were divided into two groups: the pre-vancomycin group (PVG) and the vancomycin group (VG). Statistical analyses included descriptive and comparative analyses across groups.

**Results:**

Phase One included 56 patients. Drain vancomycin concentrations peaked at POD 0, averaging 1253.67 ng/µL. Osteoblasts exposed to vancomycin concentrations of 1,250 ng/µL had significantly lower ALP activity compared to those exposed to 0, 245, 285, and 500 ng/µL (ps<0.05). Phase Two included 425 patients: 232 in the PVG and 193 in the VG. SSIs requiring debridement were observed in 3.02% of PVG patients compared to 2.07% of VG patients (p=0.761). There were no differences in pseudoarthrosis across cohorts (p=0.886), but lucency was more common in the VG (25.39%) than the PVG (11.64%; p<0.001).

**Conclusions:**

Local drain concentrations of vancomycin decreased significantly across the postoperative period, suggesting its effects on osteoblasts are limited to the early inflammatory phase. In the long-term, intrawound vancomycin may not impact risk of SSI or pseudoarthrosis.

## Introduction

In recent years, the intraoperative application of powdered vancomycin directly into the surgical wound bed has become a common, yet controversial, practice in spine surgery. This approach appeals to orthopaedic spine surgeons due to its targeted antimicrobial effect against *Staphylococcus aureus*, methicillin-resistant S*taphylococcus aureus* (MRSA), and *Staphylococcus epidermidis—*bacteria commonly responsible for surgical site infections (SSI).^1-4^ Historically, SSI prevention consisted of intravenous (IV) antibiotic prophylaxis, but infection rates following lumbar spine surgery remained as high as 13% of cases.^5,6^ Locally applied vancomycin powder has shown effectiveness in reducing SSI rates, though with some heterogeneity in the literature.^7-11^ While some studies note no change in SSI rates with intrawound vancomycin, Han et al. found that compared to a control of IV antibiotic prophylaxis alone, intrawound vancomycin decreased rates of SSI by 87.5%.^12^ There remains a concern, however, regarding vancomycin’s effects on osteoblasts and its overall effect on spinal fusion.

IV antibiotics offer broad, systemic antibiotic coverage, but cannot target specific areas of interest (i.e. surgical sites). In contrast, intrawound application of antibiotics allows for targeted prophylaxis, resulting in a high local antibiotic concentration at the surgical site.^13,14^ This may also circumvent issues with toxicity by yielding a lower systemic concentration compared to IV administration.^13,14^ However, it is imperative that the appropriate antibiotic is selected. The effects of antibiotics on osteogenesis vary by agent, and in vitro analyses have shown that several antibiotics reduce alkaline phosphatase activity, lower extracellular pH, and decrease osteoblast viability.^13,15-17^ Commonly used IV antibiotics, such as cefazolin, are associated with significant reduction in osteoblastic activity at low concentrations.^15,18^ However, Rathbone et al. reported that vancomycin was one of the least cytotoxic antibiotics tested and did not have an appreciable effect on cell viability and osteogenic activity below levels of 5,000 ng/µL.^16^ Typically, 1-2 grams of vancomycin powder is used for intrawound application—resulting in a concentration below this reported threshold. This has been confirmed by other studies, with some reporting that a vancomycin dose would need to be multiple times higher than the standard application for notable osteogenic toxicity.^18,19^ Despite its growing popularity and potential to reduce SSI, the impact of intrawound vancomycin powder on fusion integrity and biologic bone healing is not well defined. Pseudoarthrosis represents a major complication following posterior lumbar fusion (PLF), with reported rates ranging from 5-35%.^5^ However, there is little literature on long-term outcomes following the use of intrawound vancomycin after spine surgery.

This study aimed to provide data regarding the safety of vancomycin powder use in the lumbar spine in both acute and long-term periods of healing. In Phase One, an in vitro analysis was used to establish a concentration threshold at which osteoblasts are negatively impacted by vancomycin, interpreted in light of the local drain concentrations of vancomycin during the early post-operative period. The purpose of Phase One was to determine whether there is biological plausibility for vancomycin to have an effect on fusion. Then, we sought to expand upon these findings with clinical observation of potential effects of vancomycin over time. In Phase Two, retrospective cohort data was used to establish the long-term effects of vancomycin powder use on symptomatic pseudoarthrosis, as well as revisions and lucency. It was hypothesized that as vancomycin concentrations increased, metabolic activity of human osteoblasts would decrease; and, that vancomycin use at clinically relevant concentrations would be associated with reduced SSI but would have no effect on symptomatic pseudoarthrosis.

## Methods

### Phase One: in vitro Analysis

We measured drain and serum samples of patients undergoing PLF between 8/16/2012 and 1/28/2013. Upon wound closure following PLF of four or fewer levels, one gram of powdered vancomycin was placed inside the surgical site, deep to the fascia. Drain samples were collected in the post-anesthesia care unit (PACU) and daily following the procedure, beginning on post-operative day (POD) 0 and ending on POD 3. Up to four drain samples were obtained per patient. Vancomycin concentrations of these samples were analyzed, and corresponding drain outputs (and serum levels) were measured using the institution’s hospital laboratory. Average local concentrations on POD 0, 1, 2, and 3 were calculated to determine clinically relevant concentrations to be used in the in vitro analysis.

An established human osteoblast cell line (hFOB 1.19, ATCC® CRL-11372™) was utilized for the in vitro model.^17^ Cells were seeded in triplicate on tissue culture plates and fed the recommended base medium (PromoCell, C-27010) with 10% growth medium supplemental mix (PromoCell, C-39615) for 24 hours to allow attachment and establish a monolayer. After 24 hours, cell medium was changed to include vancomycin at the average concentrations determined on POD 0, 1, 2, and 3.

After 48 hours of exposure to vancomycin, samples were taken from each culture condition and processed as needed for each cellular assay. Cellular metabolic and osteogenic activities were evaluated using well-established in vitro assays including an Alamar Blue metabolic assay (Life Technologies, DAL1100) and Alkaline Phosphatase (ALP) activity kit (Abcam, ab83369).

ALP activity and cellular metabolism comparisons for different antibiotic concentrations were made using a one-way analysis of variance test, and two-group comparisons were made with student’s two-tailed t-tests. The threshold for statistical significance was p < 0.05. The JMP 11.1 statistical software suite (SAS Inc, NC) was used for statistical analysis.

### Phase Two: Retrospective Review

A retrospective chart review was conducted for all patients 18 years of age or older who underwent PLF involving four levels or fewer by spine surgeons at a single academic institution, with a minimum post-operative follow up of 1-year. One year minimum was chosen in order to adequately assess for symptomatic pseudoarthrosis, as well as to ensure adequate follow-up after early postoperative complications, including SSI.^20^ Patients were excluded if they: did not have a minimum of one year of clinical follow-up, had previous lumbar spine surgeries, were under 18 years of age, and/or had greater than four levels fused.

The Phase Two study population was divided into two groups: the pre-vancomycin group (PVG) and the vancomycin group (VG). The PVG included patients from January 1, 2007 to December 31, 2011, who were treated with the standard of care at the time: weight based IV cefazolin before the procedure and standard saline washout prior to wound closure. This institution began using intrawound vancomycin powder starting January 2012. As such, the VG included those treated from January 1, 2012, to December 31, 2015. For those in the VG, 1 gram of vancomycin was evenly spread across the surgical site wound bed deep to the fascia, in addition to standard IV antibiotic prophylaxis.

Demographic variables, surgical characteristics, and outcomes were collected via chart review. Follow up was determined by last visit to their spine surgeon for a reason relevant to the primary or revision procedures. SSIs were classified as infections requiring debridement. Pseudoarthrosis was assigned to patients by reviewing encounter notes for diagnosis of pseudoarthrosis or if they had a revision due to pseudoarthrosis. This ensured that only symptomatic (i.e., clinically relevant) pseudoarthrosis was captured. X-ray images at 1-year post-operative or the most recent imaging if greater than one year were reviewed for lucency as noted by the radiologist’s report. If a patient had a revision, the last image immediately prior to the revision was used.

Descriptive (e.g., frequencies, means, standard deviations) and comparative analyses across groups (e.g., Fisher’s exact, Chi-square, and two sample t-tests, as appropriate), in addition to logistic regressions used to control for demographic and surgical variables (where appropriate given sample sizes), were conducted using R version 4.5.1.

## Results

### Phase One

Fifty-six patients were included in Phase One. Average drain concentrations and outputs can be seen in Table 1 and Figure 1. Drain vancomycin concentrations peaked at POD 0, averaging 1,253.7 ng/µL, and decreased each subsequent day. Average drain concentrations for POD 1, 2, 3, were 497.1 ng/µL, 283.8 ng/µL, and 243.9 ng/µL, respectively. Drain output also peaked at POD 0, averaging 335.6 mL, and was lowest on POD 3 (average 71.7 mL). There were 2 patients with detectable serum concentrations of vancomycin in the PACU on POD 0, with only 20.8% of patients having detectable serum levels of vancomycin on POD 1. No patient had a detectable serum level of over 5 ng/µL. By POD 3, there were no samples with detectable levels of vancomycin.

**Table 1.** Drain Concentrations and Outputs in the Post-Operative Period

​	N	Mean	Range
Drain Concentrations (ng/μL)			
POD 0	39	1253.7	229.5-2458.2
POD 1	42	497.1	154.9-1353.1
POD 2	35	283.8	78.5-762.1
POD 3	17	243.9	28.9-617.6
Drain Output (mL)			
POD 0	50	335.6	3-930
POD 1	52	283.5	5-1245
POD 2	36	172.9	10-525
POD 3	3	71.7	30-120

**Figure 1.**
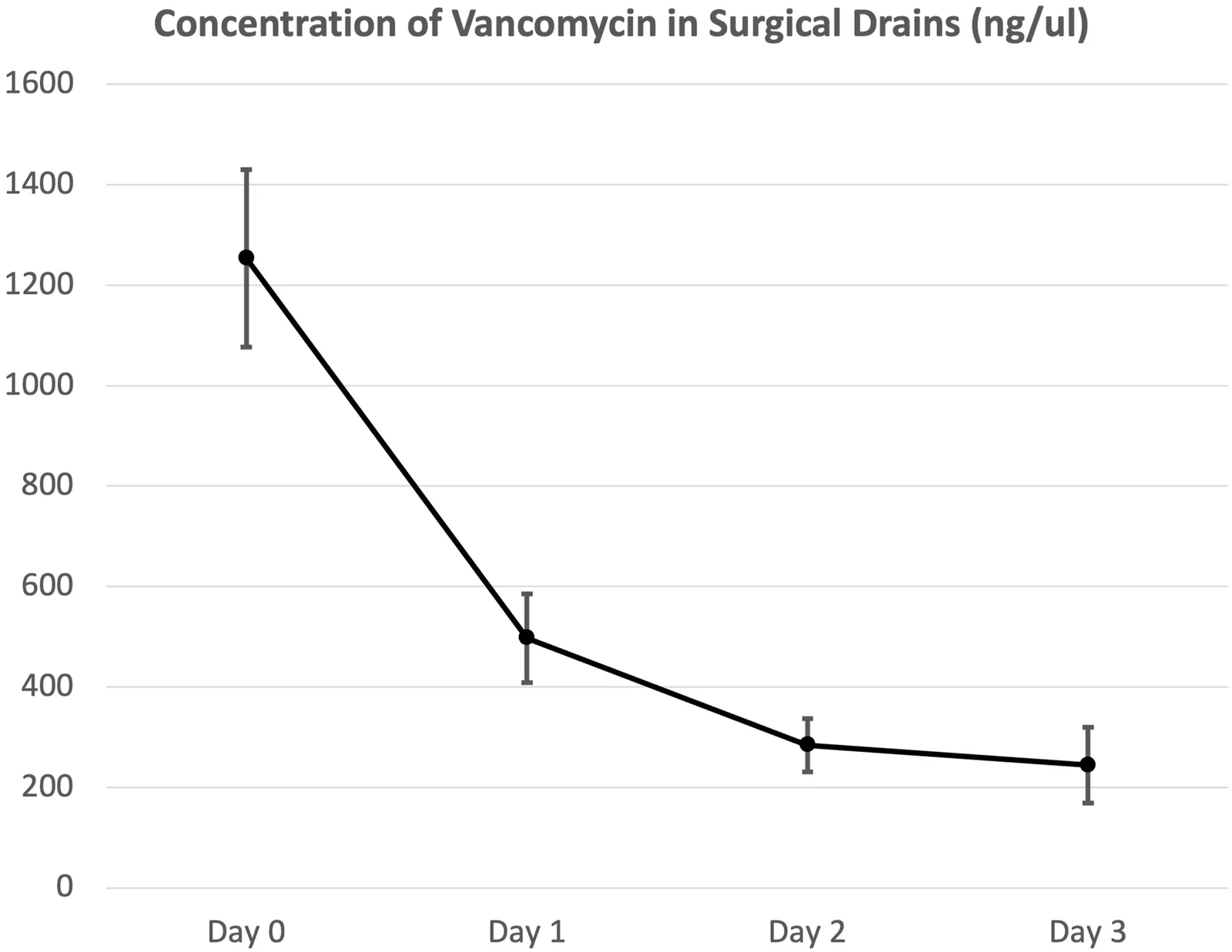
Average Concentrations of Vancomycin in Surgical Drains. Error bars represent +/- 2 standard errors

Vancomycin concentrations of 0, 245, 285, 500, and 1,250 ng/µL were selected for in vitro analysis. Figure 2 illustrates the effects of the four vancomycin concentrations on ALP and metabolic activity of human osteoblasts (HOB). Figure 2A depicts a significant difference in ALP activity with exposure of HOBs to different vancomycin concentrations (p<0.001). In particular, HOBs exposed to vancomycin concentrations of 1,250 ng/µL had significantly lower ALP activity when compared to those exposed to 0, 245, 285, and 500 ng/µL (p=0.017, p=0.004, p<0.001, and p<0.001 respectively). There was a significant increase in ALP activity when comparing HOBs exposed to vancomycin concentrations of 285 ng/µL compared to 0 ng/µL (p=0.035).

**Figure 2.**
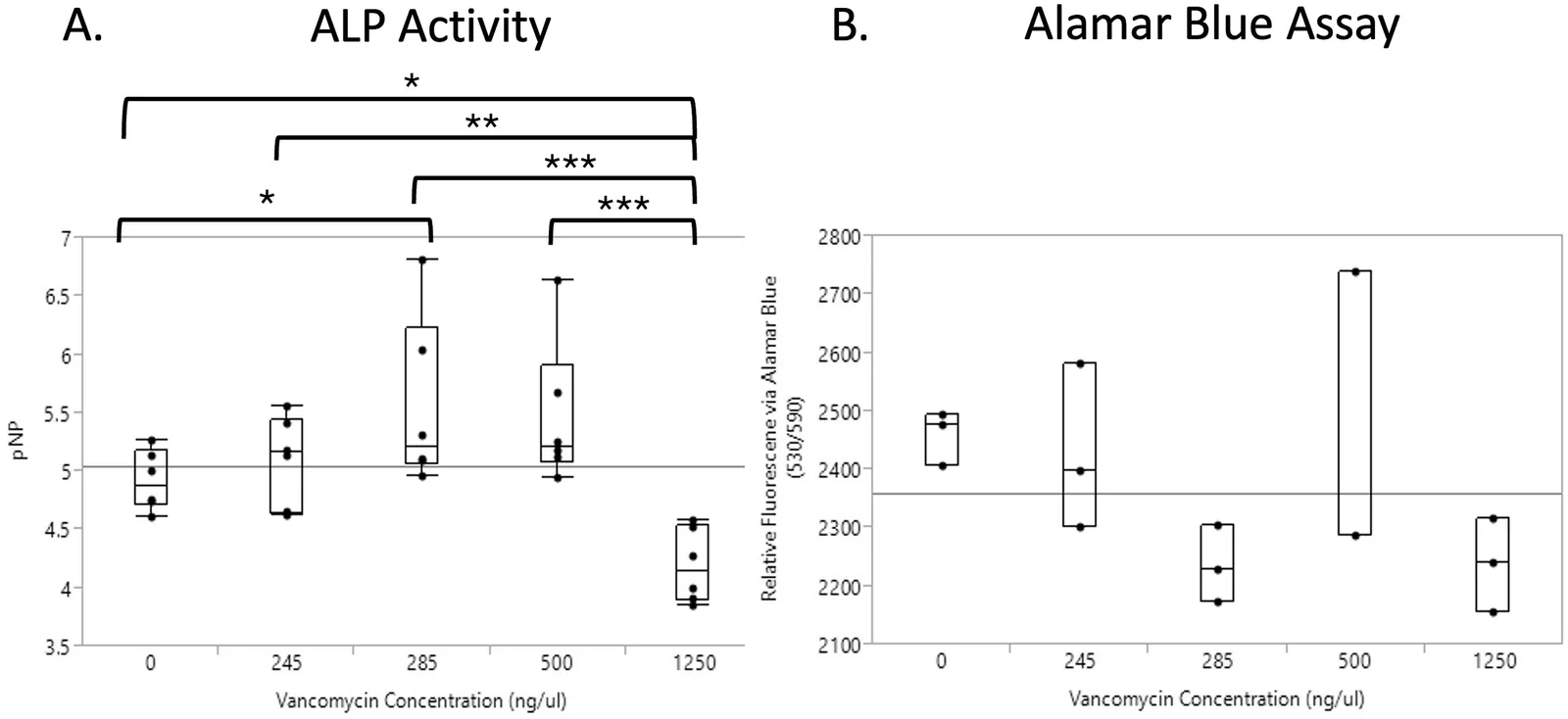
Effects of Vancomycin Concentrations on Human Osteoblasts. (A) ALP activity measured as mmol of p-nitrophenol phosphate converted to p-nitrophenol by the ALP enzyme. (B) Metabolic activity of HOB cells as a function of cellular oxidation measured by the Alamar Blue Assay. ALP: Alkaline Phosphatase; pNP: p-nitrophenol. *p<0.05, **p<0.01, ***p<0.001

Figure 2B depicts the metabolic activity of HOBs at increasing vancomycin concentrations. Metabolic activity was unaffected by the exposure to vancomycin (p=0.19).

### Phase Two

A total of 425 patients were included in Phase Two: 232 in the PVG and 193 in the VG. Baseline demographic and clinical characteristics are summarized in Table 2. Age (p<0.001) and number of levels fused (p=0.008) were significantly different across cohorts with the VG consisting of older patients and patients who were more likely to have three levels fused. All other demographic and surgical variables did not significantly differ across groups.

**Table 2.** Demographic and Surgical Characteristics of Pre-Vancomycin (PVG) and Vancomycin (VG) Groups

​	Vancomycin Powder Group				p-value
	PVG		VG		
**Sex**					0.303^c^
Female	133	57.33%	101	52.33%	
Male	99	42.67%	92	47.67%	
**Smoking Status**					0.106^c^
Nonsmoker	160	68.97%	128	66.32%	
Current Smoker	23	9.91%	11	5.70%	
Former Smoker	49	21.12%	54	27.98%	
**Diabetes Status**					0.102^f^
None	209	90.09%	160	82.90%	
Prediabetes	3	1.29%	2	1.04%	
Type I	1	0.43%	2	1.04%	
Type II	19	8.19%	29	15.03%	
**American Society of Anesthesiologists (ASA) Class**					0.478^c^
1	10	4.31%	13	6.74%	
2	140	60.34%	109	56.48%	
3	82	35.34%	71	36.79%	
**Number of Levels Involved**					**0.008** ^c^
2	131	56.47%	82	42.49%	
3	63	27.16%	78	40.41%	
4	38	16.38%	33	17.10%	
**Age**	60.30	(14.63)	65.16	(12.49)	**<0.001** ^tu^
**BMI**	28.78	(5.69)	29.30	(5.45)	0.347^t^
**Days to Follow-Up**	1765.36	(1566.15)	1739.79	(1367.70)	0.859^t^

Means (SD) for continuous variables. Frequency (%) for categorical variables.

^c^Chi-square test of association.

^f^Fisher’s exact test.

^t^t-test (equal variances).

^tu^t-test (unequal variances).

Clinical outcomes for each group are presented in Table 3, with Table 4 showing results of logistic regressions used to compare cohorts while controlling for key demographic and surgical variables (SSIs omitted from Table 4 due to insufficient statistical power). There were no differences in SSIs requiring debridement, symptomatic pseudoarthrosis, or revisions overall or in regression analyses (all p>0.05). Surgical site infections (SSIs) requiring debridement were observed in 3.02% of PVG patients compared to 2.07% of VG patients (p=0.761). Pseudoarthrosis occurred in 6.90% of the PVG and 7.25% of the VG (p=0.886). Revision surgery rates were 21.98% in the PVG and 26.94% in the VG (p=0.235). When examining reasons for revision procedures, revisions were equally likely to be due to pseudoarthrosis in the PVG (11.76%) and the VG (23.08%; p=0.194).

**Table 3.** Outcomes across Pre-Vancomycin (PVG) and Vancomycin (VG) Groups

​	Vancomycin Powder Group				p-value
	PVG		VG		
**Surgical Site Infection**					0.761^f^
No	225	96.98%	189	97.93%	
Yes	7	3.02%	4	2.07%	
**Lucency or Screw Loosening**					**<0.001** ^c^
No	205	88.36%	144	74.61%	
Yes	27	11.64%	49	25.39%	
**Pseudoarthrosis**					0.886^c^
No	216	93.10%	179	92.75%	
Yes	16	6.90%	14	7.25%	
**Revision Status**					0.235^c^
No	181	78.02%	141	73.06%	
Yes	51	21.98%	52	26.94%	

^c^Chi-square test of association.

^f^Fisher’s exact test.

**Table 4.** Logistic Regressions Predicting Outcomes from Cohort, Demographic, and Surgical Variables

​	Odds Ratio	Std. Error	Z	p-value	OR 95% CI
**Lucency or Screw Loosening**					
Intercept	0.07	1.05	-2.48	0.013	[0.01, 0.58]
Cohort (Ref=PVG)	2.47	0.28	3.25	**0.001**	[1.43, 4.25]
3 Levels (Ref=2 Levels)	1.48	0.31	1.27	0.204	[0.81, 2.70]
4 Levels (Ref=2 Levels)	3.06	0.34	3.28	**0.001**	[1.57, 5.97]
Age	1.00	0.01	0.45	0.654	[0.98, 1.03]
Days to Follow-Up	1.00	0.0001	0.55	0.582	[0.999, 1.0002]
BMI	0.99	0.02	-0.26	0.799	[0.95, 1.04]
Diabetes (Ref=None)	1.12	0.38	0.29	0.770	[0.53, 2.36]
**Pseudoarthrosis**					
Intercept	0.03	1.52	-2.30	0.021	[0.002, 0.59]
Cohort (Ref=PVG)	1.01	0.40	0.01	0.989	[0.46, 2.21]
3 Levels (Ref=2 Levels)	2.07	0.50	1.47	0.141	[0.78, 5.48]
4 Levels (Ref=2 Levels)	5.44	0.49	3.46	**<0.001**	[2.08, 14.21]
Age	0.99	0.02	-0.49	0.622	[0.96, 1.02]
Days to Follow-Up	1.0002	0.0001	2.01	**0.044**	[1.00, 1.0004]
BMI	1.01	0.03	0.18	0.859	[0.94, 1.08]
Diabetes (Ref=None)	1.28	0.55	0.45	0.656	[0.44, 3.73]
**Revision Status**					
Intercept	0.01	1.11	-4.33	<0.001	[0.001, 0.07]
Cohort (Ref=PVG)	1.47	0.28	1.39	0.166	[0.85, 2.53]
3 Levels (Ref=2 Levels)	1.37	0.31	1.00	0.315	[0.74, 2.51]
4 Levels (Ref=2 Levels)	2.18	0.36	2.15	**0.032**	[1.07, 4.42]
Age	1.00	0.01	0.22	0.828	[0.98, 1.02]
Days to Follow-Up	1.0008	0.0001	8.97	**<0.001**	[1.0006,1.001]
BMI	1.04	0.02	1.76	0.079	[0.995, 1.09]
Diabetes (Ref=None)	1.42	0.38	0.93	0.351	[0.68, 2.99]

However, screw lucency was more common in the VG (25.39%) than the PVG (11.64%; p<0.001). This difference held when controlling for demographic and surgical variables, with the odds of lucency higher in the VG than the PVG (OR=2.47, 95% CI [1.43, 4.25], p=0.001).

## Discussion

The results of this study suggest that intrawound vancomycin powder has a minimal impact on bone healing. Phase One used local drain output to establish clinically relevant concentrations of vancomycin to study the impact of local antibiotic use at the cellular level. While the local concentrations decreased rapidly during the acute post-operative period, the concentration threshold for observing a negative impact to osteoblast expression of a bone formation marker occurred as early as POD 0, well before fusion would happen in vivo, but was not sustained. Phase Two results aligned with and extended the findings of Phase One, demonstrating no impact of vancomycin use on symptomatic pseudoarthrosis in PLF patients with minimum 1-year follow-up.

The in vitro analysis aimed to elucidate the immediate effects of vancomycin on human osteoblasts. While previous studies have shown that intrawound vancomycin powder is effective in reducing infections, intrawound vancomycin’s effect on bone healing has been less widely studied. Although initial POD 0 concentrations in drain samples were found to inhibit osteoblast activity, these effects appear to be brief. Within 24 hours of surgery, surgical site vancomycin concentrations dropped to an average of 497.1 ng/µL. Edin et al. reported that vancomycin levels of up to 1,000 ng/µL at the surgical site were non-toxic and did not affect cellular replication, but others concluded that vancomycin did not have an appreciable effect on osteogenic activity at levels below 5,000 ng/µL.^15,16^ This study supports the former, as we found an inhibitory effect on osteoblastic activity at concentrations of 1,250 ng/µL. Importantly, while the average of all patients’ drain concentrations on POD 0 barely exceeded this amount, by POD 1 only one patient exceeded a concentration of 1,250 ng/µL, with no patients exceeding it by POD 2. Interestingly, osteoblastic activity appeared to increase at vancomycin concentrations of 285 ng/µL (compared to 0 ng/µL), suggesting that intrawound vancomycin powder has a temporary and non-monotonic effect on osteoblasts. This temporary effect has been previously described by Philp et al., who found that intrawound vancomycin induces a transient reduction in osteoblast proliferation that returned to control values within 72 hours.^21^ Additional studies would need to be completed to further examine these findings and eliminate any false positive that may have resulted within this small sample size.

Vancomycin concentrations at the surgical site in this study were above previously established minimal inhibitory concentrations (MIC) of *Staphylococcus aureus* strains, which range from of 1-2 ng/µL.^13^ In order to achieve these concentrations intravenously, serum levels of vancomycin are typically targeted between 15-20 ng/µL; however, this range is narrow, and vancomycin may be nephrotoxic at levels greater than 20 ng/µL.^22^ This study supports that intrawound vancomycin does not significantly enter the circulatory system, with no serum levels surpassing 5 ng/µL detected, suggesting that intrawound vancomycin is able to achieve MIC at the surgical site without increasing risk of toxicity for the patient.

Our results also indicate a rapid decrease in surgical site concentrations of vancomycin by POD 1, with smaller decreases to POD 2 and POD 3. By POD 3, average concentrations of vancomycin had no effect on ALP nor metabolic activity of osteoblasts. At no concentration investigated during this study did vancomycin affect osteoblast metabolic activity. Therefore, it appears that vancomycin is metabolized during the inflammatory phase of healing, which typically lasts about 5 days. As such, vancomycin should not impair the repair and remodeling stages of bone healing. Additionally, initial union following spine fusion occurs by around 4-6 weeks, with adequate strength achieved by around 6 months.^23,24^ In summary, although there was a measurable effect on osteoblast expression of this biochemical marker of bone formation in the immediate post-operative period, it may not be significant enough to clinically alter bone healing over the long-term.

To analyze these long-term effects of intrawound vancomycin powder on bone healing following PLF, the second phase of our study consisted of a retrospective analysis. The use of intrawound vancomycin powder in short-segment PLF was not significantly associated with pseudoarthrosis, revision, or SSI rates. Symptomatic pseudoarthrosis rates for both groups were nearly identical, indicating that intrawound vancomycin has no association with diagnosed pseudoarthrosis. There was increased screw lucency or loosening on radiographic reads in the VG, which held when controlling for demographic and surgical differences between cohorts. It is possible that this increased lucency in the VG represents patients who have asymptomatic pseudoarthrosis; however, it is also possible that these patients went on to fuse following initial instrumentation loosening.^25-27^ In addition, this finding may be explained by differences in patient populations, such as the presence of osteoporosis or unmeasurable factors; or, it may simply represent changes in imaging techniques and reporting between the two study time periods due to the use of a historical-control design.^25^

No difference was found between cohorts in SSI rates requiring debridement; however, the current analysis was limited by only including 11 total cases of SSIs across both cohorts. Current literature demonstrates conflicting conclusions regarding the efficacy of intrawound vancomycin in preventing post-operative infection after spine surgery. Our results contrast with prior literature concluding that there is a decrease in SSI rates when utilizing intrawound vancomycin powder.^1,3,28-31^ However, our results of a large sample size with long-term follow-up support more recent literature stating that intrawound vancomycin is not associated with reduced SSI rates in spinal surgery.^32-34^ These conflicting results emphasize the importance of randomized controlled trials to determine the role of intraoperative vancomycin in open spine surgery.

Revision rates were not significantly different between groups, likely due to the multifactorial causes of reoperation, including hardware failure, adjacent segment disease (ASD), and other complications not directly related to infection or fusion status. We examined causes of revision rates within the sample: in both groups, revisions were largely due to ASD and stenosis. Revisions for symptomatic pseudoarthrosis were equally common in the PVG and the VG—further supporting that vancomycin does not affect fusion rates.

This study has several limitations. There is a possibility of both systematic and random errors during Phase One sample collection. Additionally, the use of a homogenized, established fetal osteoblast cell line may not be representative of the population’s mature cellular response. Future work in this area could use cellular samples isolated directly from the surgical site. While local drain concentrations showed a decline, this is likely not indicative of exposure to the antibiotic powder at the cellular level when delivered directly to the tissue/cells. Because the actual concentration experienced by the cells in vivo has not been adequately characterized, the decision was made to keep the concentration static for in vitro investigation.

Furthermore, the retrospective design in Phase Two, in particular the use of historical controls, introduces several potential sources of bias. There may be unmeasured changes in surgical and imaging techniques, perioperative protocols and reporting, and postoperative care over time that are confounded with vancomycin use. This allows for alternative explanations for the results, including the finding that there was increased lucency in the VG; for example, rather than representing true differences in screw loosening due to vancomycin application, the significant result could be a byproduct of temporal changes in imaging technique and reporting. Such an explanation would align well with the lack of significant differences in symptomatic pseudoarthrosis cases across cohorts, and therefore cannot be ruled out as a plausible alternative explanation.

Additionally, definitions used for key variables in this study may limit its generalizability. For example, because SSI was defined as patients requiring debridement, rates reported in this research may be biased by treating surgeons’ clinical assessments and other factors impacting their decisions. Also, radiographic imaging was used to determine screw lucency, rather than CT (computer tomography) diagnostic imaging. In this study, only 50% of positive pseudoarthrosis cases had a confirmatory CT. Thus, the overall rate of asymptomatic pseudoarthrosis in our sample may be under- or overestimated relative to the true incidence of the diagnosis.

Finally, Phase Two aimed to assess the long-term effects of vancomycin during the first two years of its implementation as standard of care at a single institution. As such, the sample size, particularly in the VG, may be underpowered to detect small differences in low-incidence outcomes such as SSI. Beyond issues of statistical power, rates of complications at the first introduction of a new protocol (in this case, vancomycin) might not be comparable to rates of complications when longer-term protocols were being used (as in the pre-vancomycin group).

The findings of this study indicate that although intrawound vancomycin may have an effect on osteoblast activity during the immediate post-operative period (e.g., POD 0), there are few significant differences in long-term outcomes when compared to patients who did not receive intrawound vancomycin treatment. Our findings suggest that the immediate and temporary post-operative inhibition of osteoblasts may be time-limited, as we found no long-term impact on pseudoarthrosis. However, broader implications must also be considered, especially in light of nonsignificant differences in SSI rates between cohorts. Routine intraoperative antibiotic use raises concerns regarding microbial resistance, altered microbiomes, seroma, and unintended effects on surrounding tissues. Developing evidence-based guidelines for vancomycin powder application is essential to balance infection prevention with bone health and long-term patient safety.

Intrawound vancomycin was associated with transient, concentration-dependent changes in osteoblast ALP activity in vitro; however, these effects are limited to the inflammatory phase of healing when fusion is unlikely to be impacted. In the historical-control cohort of posterior lumbar fusion patients, vancomycin use was, in fact, not associated with lower rates of infection requiring debridement, symptomatic pseudoarthrosis, or revision, but was associated with higher rates of radiographic screw lucency. Prospective studies are needed to clarify whether this lucency signal reflects true biologic effect or residual confounding.

## Data Availability

Data will be made available upon reasonable request.*
